# Comparative Analysis of Six Adeno-Associated Viral Vector Serotypes in Mouse Inferior Colliculus and Cerebellum

**DOI:** 10.1523/ENEURO.0391-24.2024

**Published:** 2024-11-05

**Authors:** Isabelle Witteveen, Timothy Balmer

**Affiliations:** School of Life Sciences, Arizona State University, Tempe, Arizona 85287

**Keywords:** adeno-associated virus, cerebellum, inferior colliculus

## Abstract

Adeno-associated viral vector (AAV) serotypes vary in how effectively they express genes across different cell types and brain regions. Here we report a systematic comparison of the AAV serotypes 1, 2, 5, 8, 9, and the directed evolution derived AAVrg, in the inferior colliculus (IC) and cerebellum. The AAVs were identical apart from their different serotypes, each having a synapsin promotor and expressing GFP (AAV-hSyn-GFP). Identical titers and volumes were injected into the IC and cerebellum of adult male and female mice, and brains were sectioned and imaged 2 weeks later. Transduction efficacy, anterograde labeling of axonal projections, and retrograde labeling of somata were characterized and compared across serotypes. Cell-type tropism was assessed by analyzing the morphology of the GFP-labeled neurons in the cerebellar cortex. In both the cerebellum and IC, AAV1 expressed GFP in more cells, labeled a larger volume, and produced significantly brighter labeling than all other serotypes, indicating superior transgene expression. AAV1 labeled more Purkinje cells, unipolar brush cells, and molecular layer interneurons than the other serotypes, while AAV2 labeled a greater number of granule cells. These results provide guidelines for the use of AAVs as gene delivery tools in these regions.

## Graphical abstract

**Figure:**
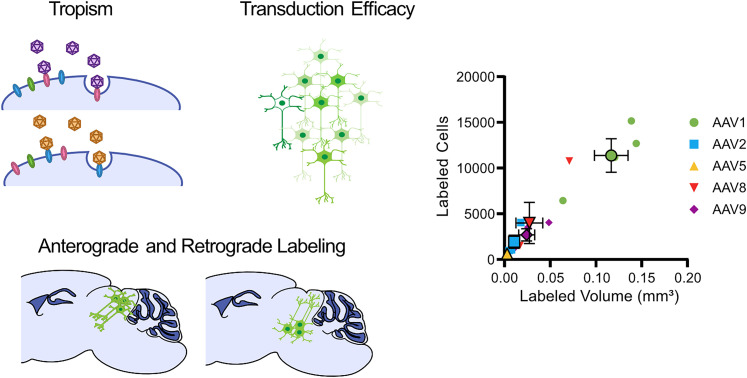
Visual Abstract

## Significance Statement

AAVs have become ubiquitous gene expression tools in neuroscience research and are becoming more common in clinical settings. Naturally occurring and engineered serotypes have varying abilities to infect neurons and cause them to produce proteins of interest. The efficacy of AAV transduction in specific cell types depends on many factors and remains difficult to predict, so an empirical approach is often required to determine the best performing serotype in each population of cells. In the present study, we show that AAV1 produces the highest expression in these two regions, labels the most axonal projections, and labels Purkinje cells and unipolar brush cells better than the other serotypes tested, while AAV2 labels granule cells most effectively.

## Introduction

Adeno-associated viruses (AAVs) have become critical tools in neuroscience research and hold substantial promise in clinical gene therapy applications. AAVs have been developed to treat some genetic disorders that result from impaired gene expression, including specific forms of inherited retinal disease and deafness (D. Wang et al., 2019; Haery et al., 2019; Haggerty et al., 2020; Xue et al., 2023; Lv et al., 2024). AAVs are small, ∼24 nm, viruses belonging to the *Dependoparvovirus* genus, named for their inability to self-replicate without the presence of helper viruses such as Adeno virus (Atchison et al., 1965; Hoggan et al., 1966; D. Wang et al., 2019; Haery et al., 2019; Haggerty et al., 2020). Despite their prevalence in the human population, 40–80% of humans are seropositive for antibodies against AAVs—they are not known to cause any human disease (D. Wang et al., 2019). Their low immunogenicity and ability to induce long-term gene expression in postmitotic cells have made AAVs attractive candidate vectors for use in gene therapy (Snyder and Moullier, 2011; D. Wang et al., 2019; Haery et al., 2019; Haggerty et al., 2020).

AAVs have become widely adopted tools for gene expression in neuroscience research. For example, AAVs are routinely used to express fluorescent reporters for the visualization and mapping of neuronal circuits. The delivery of Cre and other recombinases allows conditional gene expression or deletion to examine the functions of proteins, cell types, and circuits. The expression of optogenetic and chemogenetic tools can be used to control the activity of neurons. The delivery of calcium- and voltage-sensitive fluorescent indicators allows the visualization of neuronal activity. Many more tools are available and continue to be developed through the use of AAVs (Yizhar et al., 2011; Haery et al., 2019). Given their utility, analyses of the compatibility of different AAV serotypes in specific brain regions have become essential.

A variety of naturally occurring AAV serotypes have been found, each with varying capsid proteins and genomes (Snyder and Moullier, 2011; D. Wang et al., 2019). The effectiveness of AAV transduction is determined by interactions between capsid proteins and cell surface receptors, internalization, and subsequent downstream effects (Snyder and Moullier, 2011; D. Wang et al., 2019). Since serotypes recognize distinct receptors, this leads to species-specific, tissue-specific, and cell-type–specific tropism (Snyder and Moullier, 2011; D. Wang et al., 2019). Pseudotyping takes advantage of this natural tropism by packaging the recombinant AAV genome into the capsids of different serotypes to increase transduction efficacy (Burger et al., 2004). Numerous studies have since characterized differences in AAV transduction between serotypes in various brain regions, including the nigrostriatal system, hippocampus, cerebral cortex, and a variety of other midbrain regions, utilizing both local and systemic injections (C. Wang et al., 2003; Burger et al., 2004; Paterna et al., 2004; Cearley and Wolfe, 2006; Taymans et al., 2007; Klein et al., 2008; Zincarelli et al., 2008; McFarland et al., 2009; Blits et al., 2010; Mason et al., 2010; Hutson et al., 2012; Aschauer et al., 2013; Holehonnur et al., 2014; Watakabe et al., 2015). These studies demonstrate the presence of region-specific tropism and underscore the necessity of empirical comparisons of AAV serotypes in well-controlled experiments. Quantitative analyses of the transduction efficacy of AAV serotypes in the inferior colliculus (IC) and cerebellum have not been reported.

The cerebellum is a structure necessary for coordinated movement and has been increasingly implicated in cognitive functions, while the IC is a major processing center of ascending and descending auditory pathways (Apps and Garwicz, 2005; Winer and Schreiner, 2005; Reeber et al., 2013; De Zeeuw et al., 2021). Previous research has utilized AAVs in these regions, but few have reported the effectiveness of different AAV serotypes. The transduction patterns of Purkinje cells have been examined for some serotypes (Kaemmerer et al., 2000; Hirai, 2008; Bosch et al., 2014; Kim et al., 2015; El-Shamayleh et al., 2017). Other studies have compared the transduction efficacy of some AAV serotypes at early postnatal ages (Broekman et al., 2006), differences in short-term synaptic plasticity following AAV-mediated channelrhodopsin expression (Jackman et al., 2014), or transduction patterns of an individual AAV serotype (Alisky et al., 2000). The goal of this study was to characterize the transduction and tropism of commercially available AAV serotypes following in vivo injection into the IC and cerebellum, which can inform further utilization of AAVs in these regions.

## Materials and Methods

### Animals

C57BL6/J mice of both sexes were used in this study (17 males, 15 females). The ages ranged from 2 to 3 months old. Mice were bred in a colony maintained in the animal facility managed by the Department of Animal Care and Technologies, and all procedures were approved by Arizona State University Institutional Animal Care and Use Committee under protocol #21-1817R.

### Adeno-associated viruses

All viruses were purchased from Addgene containing EGFP under the control of the human synapsin promoter (Addgene plasmid # 50465; RRID: Addgene_50465). The serotypes included AAV1, AAV2, AAV5, AAV8, AAV9, and AAVrg. AAVrg was engineered to infect axons (Tervo et al., 2016), in addition to somata at the injection site. Images of labeling with AAVrg are shown in some figures, although this serotype was only included in the statistical comparisons of cell-type tropism in the cerebellar cortex. Aliquots of 5 µl were stored at −80°C. Identical titers were used across all injections, 8 × 10^12^ GC/ml (4 × 10^9^ GC per 500 nl injection). Aliquots were thawed on ice and diluted with sterile saline to achieve this titer on the day of injection.

### Viral injections

Viral injections were made into the cerebellum and the IC under isoflurane anesthesia. Glass capillaries were pulled on a horizontal puller and beveled at a 45° angle to a 20–30 µm inside diameter. The glass pipettes were filled with mineral oil and placed on the injector (Nanoliter2020, World Precision Instruments). Mice were weighed and then anesthetized in a knockdown chamber at a flow rate of 500 ml/min with 5% isoflurane and then placed in a stereotax with the flow rate reduced to 50 ml/min with 1–2% isoflurane, and adjustments were made based on the frequency of respiration of the mouse using a digital vaporizer (SomnoSuite, Kent Scientific). Ophthalmic lubricant was applied to the eyes of the mouse to prevent drying. Following fur removal, the scalp was cleaned using 7.5% povidone iodine. An incision was made in the scalp and one or two small holes were drilled in the skull with a burr drill. Virus was placed on parafilm and then taken up into the pipette. The pipette was slowly lowered into the brain at a rate of ∼10 µm/s. Three minutes after the pipette reached the injection site 500 nl of virus was injected at 5 nl/s. Three minutes after the injection the pipette was slowly retracted at a rate of 10 µm/s. Injections were made at the stereotaxic coordinates of 7.2 mm caudal, 0.5 mm lateral to bregma, and 2.9 mm ventral to the surface of the brain for the cerebellum and 5.5 mm caudal, 1.1 mm lateral to bregma, and 1.0 mm ventral to the surface of the brain for the right IC.

### Immunohistochemistry and imaging

Two weeks after injection mice were overdosed with isoflurane and perfused through the heart with 0.01 M phosphate-buffered saline (PBS), pH 7.4, followed by 4% paraformaldehyde in PBS. Brains were extracted from the skull and incubated in 4% paraformaldehyde in PBS overnight. Brains were suspended in 3% agarose in PBS and sectioned in ice-cold PBS with a vibratome (7000smz-2, Campden Instruments). Then, 50 µm sections were collected and placed free-floating in well plates filled with PBS. Every fourth section was stained with DAPI in PBS (1:2,000) for 1 h, washed three times for 5 min in PBS, mounted on microscope slides (Superfrost Plus, Fisher Scientific), and coverslipped with Fluoromount-G (SouthernBiotech). Slides were imaged using an Olympus VS200 Slide Scanner with a 20× objective using an X-Cite XYLIS (Excelitas Technologies) broad-spectrum LED light that was maintained at 100% power. Cerebellum injection sites and the axonal projections from both cerebellum and IC were imaged using an exposure time of 1.493 ms for DAPI (378/52 nm excitation filter, 432/36 nm emission filter), and 16.617 ms exposure time for GFP (474/27 nm excitation filter, 515/30 emission filter). Many images of the IC injection site were saturated with these exposure times, so IC was also imaged with a reduced exposure time of 2.53 ms for GFP. Additional 63× images were acquired using a Zeiss LSM 800 confocal microscope.

### Image analysis

To address variations in background fluorescence in the 20× slide scanner images, background subtraction and thresholding functions were used in FIJI (Schindelin et al., 2012). Regions of interest were made and watershed and analyze particle functions in FIJI were used to count the number of labeled somas within the IC. All labeled cells in the cerebellum were counted using the same functions including both the cerebellar cortex and cerebellar nuclei. Labeled volume is a measure that represents a combination of labeling density and distance of spread and was calculated as the number of pixels above a brightness threshold of 20 across all collected slices. Each pixel was 0.325 µm^2^, the depth was the thickness of the slice (50 µm), and the final volume was multiplied by four to account for imaging every fourth slice. Mean pixel brightness was calculated as the average of the pixel intensities that were above threshold in the image.

The ability of the AAV serotypes to label different cell types in the cerebellar cortex was analyzed in FIJI (Schindelin et al., 2012). Regions of interest were created using the wand tool to outline individual labeled cells, and cell types were annotated and counted based on their morphology. Purkinje cells were identified by their large soma size, extensive dendritic tree, and location in the Purkinje cell layer. Unipolar brush cells (UBCs) were identified by their location in the granule cell layer and unique brush dendrite. Golgi cells were identified by their large size and presence in the granule cell layer. Granule cells were determined by their small size, absence of dendritic brush, and location in the granule cell layer. The labeling intensity varied across granule cells from bright to quite dim across serotypes. Some labeled granule cells were not markedly brighter than the surrounding neuropil (Kim et al., 2015). However, the observation that many DAPI-positive cells were completely devoid of any GFP fluorescence (Fig. 3*B*, white arrows) justified identifying these dimly labeled cells as expressing the transgene. There was no apparent difference in the proportion of brightly labeled granule cells across serotypes. Molecular layer interneurons were determined by their small size and presence in the molecular layer. Nine to 14 images were analyzed for each serotype using 2–4 images (567.90 µm × 567.90 µm) per animal (four animals for AAV1, 2, 5, 8, and 9 and three animals for AAVrg) per serotype.

To analyze retrograde labeling for each serotype, the number of labeled cell bodies in each region was counted manually, using the Allen Institute Mouse Brain Atlas as a reference. To analyze anterograde axonal projections, the background subtraction function in FIJI was applied and the total number of pixels above the threshold was used as an estimate of the density of labeled axons. One image per animal per region (3–4 per serotype) was analyzed for each brain region for both retrograde and anterograde labeling studies.

Identical imaging parameters and brightness and contrast adjustments were applied to all images within each figure panel that were included in quantitative comparisons. Nonlinear adjustments (gamma) were not applied to any image.

### Statistics

The Shapiro–Wilk test was used to determine whether the data were normally distributed. Groups were compared using one-way ANOVAs followed by Tukey's HSD tests. Statistical analyses are not reported for data where more than two of the serotypes failed the Shapiro–Wilk test and instead qualitative comparisons are reported. Prism (GraphPad) and R were used for statistical tests and for generating figures.

## Results

The aim of this study was to test the efficacy of different AAV serotypes in transducing neurons in the IC and cerebellum of mice. Identical titers (8 × 10^12^ GC/ml) and volumes (500 nl) of AAV-hSyn-GFP constructs in the AAV serotypes 1, 2, 5, 8, 9, and the directed evolution derived AAVrg (Tervo et al., 2016) were stereotaxically injected into the brains of anesthetized mice. The mice quickly recovered from the procedure and did not show any changes in behavior or health during the 2 week incubation period. For each region, at least four animals were used for each serotype. Following transcardiac perfusion, brain extraction, and fixation, 50 µm serial sections were collected, and every fourth section was stained with DAPI, mounted on slides, imaged, and analyzed.

### Efficacy of transgene expression in the IC

**Figure 1.**
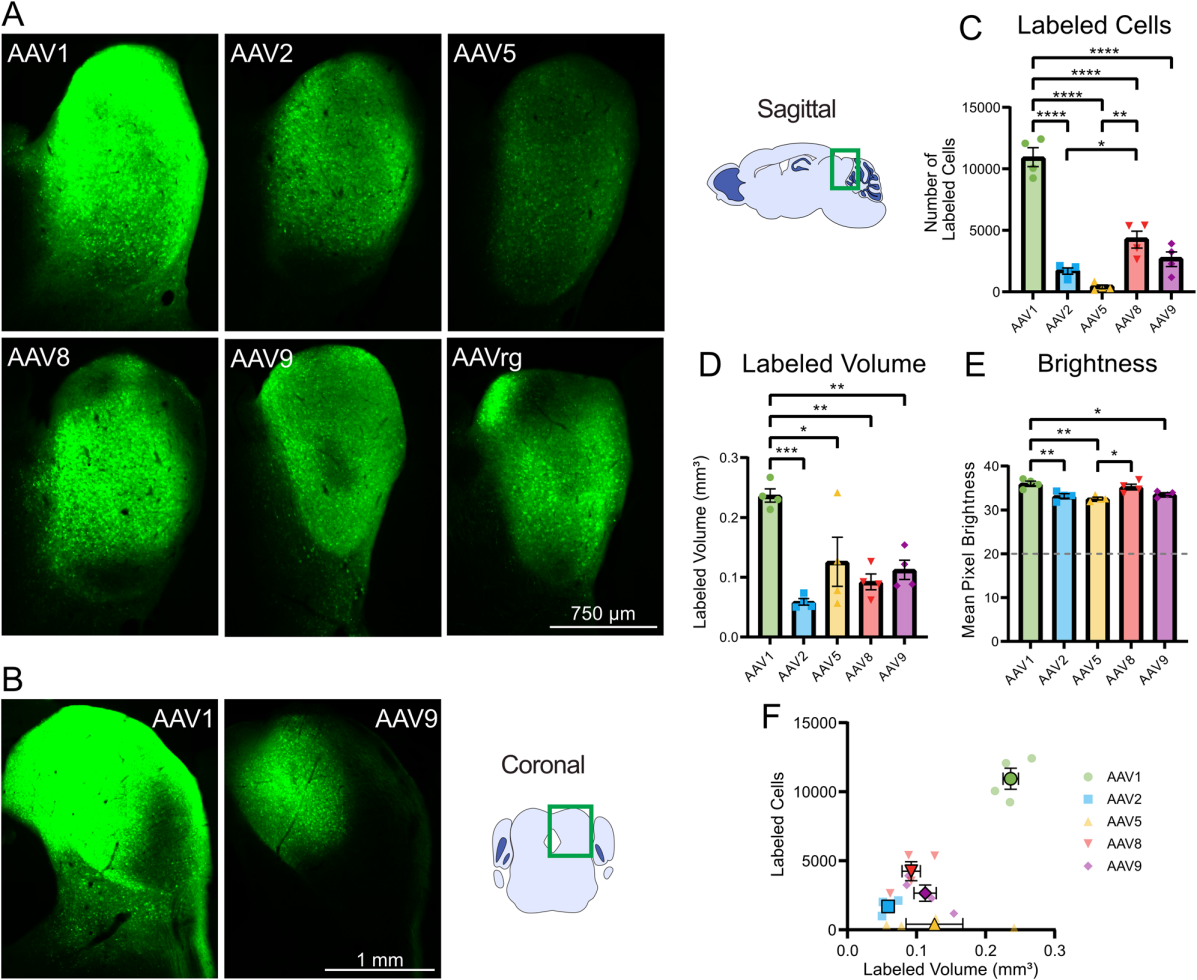
AAV1 produced the highest transgene expression and the largest labeled volume in the IC. ***A***, Intracranial injection of the AAV1, 2, 5, 8, 9, and rg serotypes led to GFP expression in the IC. Representative sagittal sections of the injection site in the IC for each serotype. ***B***, Additional representative coronal sections of AAV1 and AAV9 injected IC. ***C***, Bar graph showing mean number of labeled cells across serotypes. AAV1 labeled significantly more cells than AAV2, 5, 8, and 9, indicating superior transduction efficacy. ***D***, Bar graph showing mean volume of labeling across serotypes. The volume of AAV1 labeling also was significantly greater than that of AAV2, 5, 8, and 9. Horizontal line indicates the brightness threshold over which pixels were considered labeled and were included in the mean brightness calculation. ***E***, Bar graph showing mean pixel brightness across serotypes. AAV1 produced significantly greater mean pixel brightness than AAV2, 5, and 9. ***F***, Comparison of number of labeled cells with labeled volume. Identical imaging parameters and brightness and contrast adjustments were applied to all images in the same panel. Statistical comparisons were made using one-way ANOVAs followed by Tukey's HSD tests. Error bars are SEM. * indicates *p* < 0.05, ** indicates *p* < 0.01, *** indicates *p* < 0.001, **** indicates *p* < 0.0001.

First, we tested the ability of the most widely available AAV serotypes to drive transgene expression in the IC. The AAV injections were made into the central IC and the volume of 500 nl was sufficient to diffuse to all regions including the dorsal and external nuclei. GFP was expressed by all serotypes, in all experiments (Fig. 1*A*). In some figures, the serotype AAVrg is shown for comparison but is not included in statistical analyses of transduction volume and brightness. AAV1 drove the highest GFP expression in the IC compared with the other serotypes with a substantial degree of spread (Fig. 1*A*,*B*), while AAV5 had the lowest GFP expression (Fig. 1*A*). To quantitatively compare the transduction efficacy across serotypes, we calculated the total number of labeled cells within the IC of the imaged sections. Significantly more cells were labeled by AAV1 than AAV2, 5, 8, and 9 (Fig. 1*C*, Table 1). Additionally, AAV8 showed a significantly greater number of labeled cells than AAV2 and 5 (Fig. 1*C*). AAV1 labeled more cells outside the IC in the adjacent periaqueductal gray than all other serotypes (one-way ANOVA; *F*_(4,15)_ = 16.44; *p* < 0.0001; Tukey's HSD tests; AAV1 vs AAV2, 5, 8, *p* < 0.001; AAV1 vs AAV9, *p* = 0.0009). Although AAV9 labeled far fewer cells in IC than did AAV1, the percentage of cells labeled outside the IC of the total population was similar between them, indicating similar spread (AAV1, 10.6 ± 1.98%; AAV9, 11.0 ± 2.78%), while the other AAVs labeled few cells outside IC resulting in labeling that was more restricted to the injection site (AAV2, 3.6 ± 1.0%; AAV5, 1.9 ± 0.4%; AAV8, 1.6 ± 1.2%; mean ± SEM). The volume of viral labeling was determined by summing the total number of GFP-positive pixels across all slices. AAV1 transduced a significantly greater volume compared with AAV2, 5, 8, and 9 (Fig. 1*D*). Finally, the mean pixel brightness for each serotype was calculated, showing cells infected with AAV1 had significantly brighter GFP expression compared with those infected with AAV2, 5, and 9 (Fig. 1*E*). In sum, AAV1 labeled the most IC neurons and labeled the largest volume, while serotypes AAV2, 5, 8, and 9 were far less effective (Fig. 1*F*).

**Table 1.** Statistics associated with Figure 1

Associated figure	Statistical test	Groups compared	Test statistic	*p* value	Mean difference	*N* (animals)
Fig. 1*C*	One-way ANOVA	All	*F*_(4,15)_ = 56.76	<0.0001		20
Fig. 1*C*	Tukey's test	AAV1 vs AAV2	*q* = 16.87	<0.0001	9,260	8
Fig. 1*C*	Tukey's test	AAV1 vs AAV5	*q* = 19.21	<0.0001	10,547	8
Fig. 1*C*	Tukey's test	AAV1 vs AAV8	*q* = 12.22	<0.0001	6,707	8
Fig. 1*C*	Tukey's test	AAV1 vs AAV9	*q* = 15.11	<0.0001	8,294	8
Fig. 1*C*	Tukey's test	AAV5 vs AAV8	*q* = 6.993	0.0014	−3,840	8
Fig. 1*C*	Tukey's test	AAV2 vs AAV8	*q* = 4.650	0.0343	−3,840	8
Fig. 1*D*	One-way ANOVA	All	*F*_(4,15)_ = 9.842	0.0004		20
Fig. 1*D*	Tukey's test	AAV1 vs AAV2	*q* = 8.301	0.0003	0.1775	8
Fig. 1*D*	Tukey's test	AAV1 vs AAV5	*q* = 5.161	0.0172	0.1104	8
Fig. 1*D*	Tukey's test	AAV1 vs AAV8	*q* = 6.736	0.0020	0.1441	8
Fig. 1*D*	Tukey's test	AAV1 vs AAV9	*q* = 5.792	0.0072	0.1239	8
Fig. 1*E*	One-way ANOVA	All	*F*_(4,15)_ = 8.588	0.0008		20
Fig. 1*E*	Tukey's test	AAV1 vs AAV2	*q* = 5.759	0.0076	2.836	8
Fig. 1*E*	Tukey's test	AAV1 vs AAV5	*q* = 6.963	0.0015	3.429	8
Fig. 1*E*	Tukey's test	AAV1 vs AAV9	*q* = 4.943	0.0231	2.434	8
Fig. 1*E*	Tukey's test	AAV5 vs AAV8	*q* = 5.350	0.0133	−2.635	8

### Efficacy of transgene expression in the cerebellum

**Figure 2.**
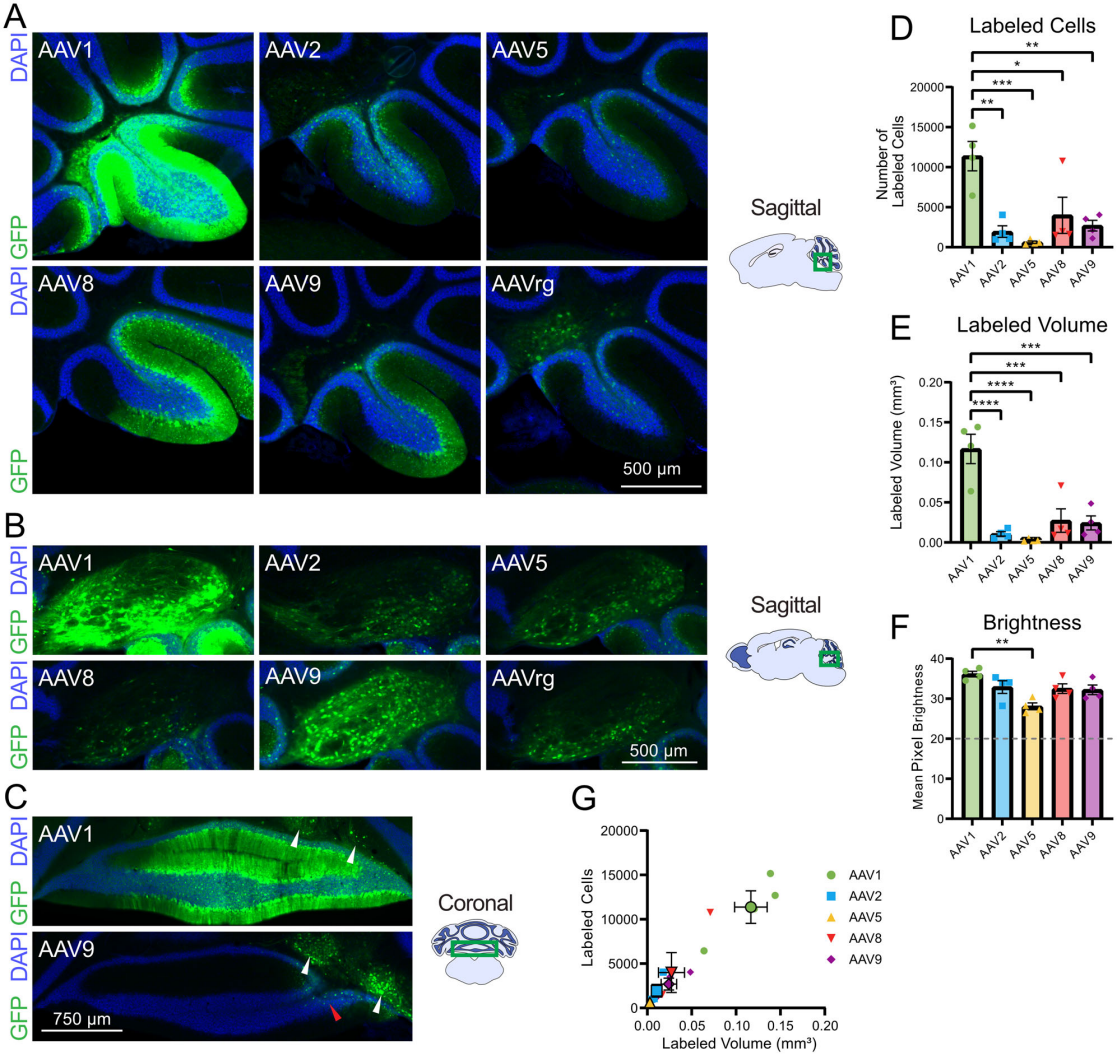
AAV1 produced the highest transgene expression and largest labeled volume in the cerebellum. ***A***, Representative sagittal sections of injected cerebellar cortex for each serotype. ***B***, Representative sagittal sections of the interposed nucleus. ***C***, Additional representative coronal sections of AAV1 and AAV9 injected cerebellum. White arrows indicate transfected cells in the deep cerebellar nuclei, and the red arrow indicates the minimal spread to the cerebellar cortex by AAV9. ***D***, Bar graph showing mean number of labeled cells across serotypes. AAV1 labeled significantly more cells than AAV2, 5, 8, and 9, indicating superior transduction efficacy. ***E***, Bar graph showing mean volume of labeling across serotypes. The labeled volume of AAV1 also was significantly greater than that of AAV2, 5, 8, and 9. ***F***, Bar graph showing mean pixel brightness across serotypes. AAV1 produced significantly greater mean pixel brightness compared with AAV2, 5, 8, and 9. Horizontal line indicates the brightness threshold over which pixels were considered labeled and were included in the mean brightness calculation. ***G***, Comparison of number of cells labeled by labeled volume. Identical imaging parameters and brightness and contrast adjustments were applied to all images in the same panel. Statistical comparisons were made using one-way ANOVAs followed by Tukey's HSD tests. Error bars are SEM. * indicates *p* < 0.05, ** indicates *p* < 0.01, *** indicates *p* < 0.001, **** indicates *p* < 0.0001.

These AAVs were also tested in the cerebellum, where the injections were focused on the lateral aspect of lobule X. We chose this region because lobule X is part of the vestibulocerebellum and contains a high density of UBCs, in addition to the other cell types present throughout all lobes of the cerebellar cortex. Additionally, the lateral injection site allowed for transfection of the cerebellar nuclei. AAV1 labeled significantly more cerebellar neurons than did AAV2, 5, 8, and 9 (Fig. 2*A–D*, Table 2). The volume of brain labeled by AAV1 was also significantly greater than that of AAV2, 5, 8, and 9 (Fig. 2*E*). Comparing the mean pixel brightness across serotypes revealed that AAV1 labeling was brighter than the other serotypes and reached significance compared with AAV5 (Fig. 2*F*). Thus, as in the IC, AAV1 labeled the most neurons and labeled the largest volume while the other serotypes produced much more restricted expression (Fig. 2*G*).

**Table 2.** Statistics associated with Figure 2

Associated figure	Statistical test	Groups compared	Test statistic	*p* value	Mean difference	*N* (animals)
Fig. 2*D*	One-way ANOVA	All	*F*_(4,15)_ = 9.440	0.0005		20
Fig. 2*D*	Tukey's test	AAV1 vs AAV2	*q* = 6.843	0.0017	9,418	8
Fig. 2*D*	Tukey's test	AAV1 vs AAV5	*q* = 7.808	0.0005	10,746	8
Fig. 2*D*	Tukey's test	AAV1 vs AAV8	*q* = 5.366	0.0130	7,384	8
Fig. 2*D*	Tukey's test	AAV1 vs AAV9	*q* = 6.300	0.0036	8,671	8
Fig. 2*E*	One-way ANOVA	All	*F*_(4,15)_ = 16.65	<0.0001		20
Fig. 2*E*	Tukey's test	AAV1 vs AAV2	*q* = 9.408	<0.0001	0.1062	8
Fig. 2*E*	Tukey's test	AAV1 vs AAV5	*q* = 10.09	<0.0001	0.1139	8
Fig. 2*E*	Tukey's test	AAV1 vs AAV8	*q* = 7.938	0.0004	0.08958	8
Fig. 2*E*	Tukey's test	AAV1 vs AAV9	*q* = 8.199	0.0003	0.09252	8
Fig. 2*F*	One-way ANOVA	All	*F*_(4,15)_ = 6.205	0.0037		20
Fig. 2*F*	Tukey's test	AAV1 vs AAV5	*q* = 6.999	<0.0001	8.064	8

### Analysis of cell-type tropism for AAV serotypes in the cerebellum

**Figure 3.**
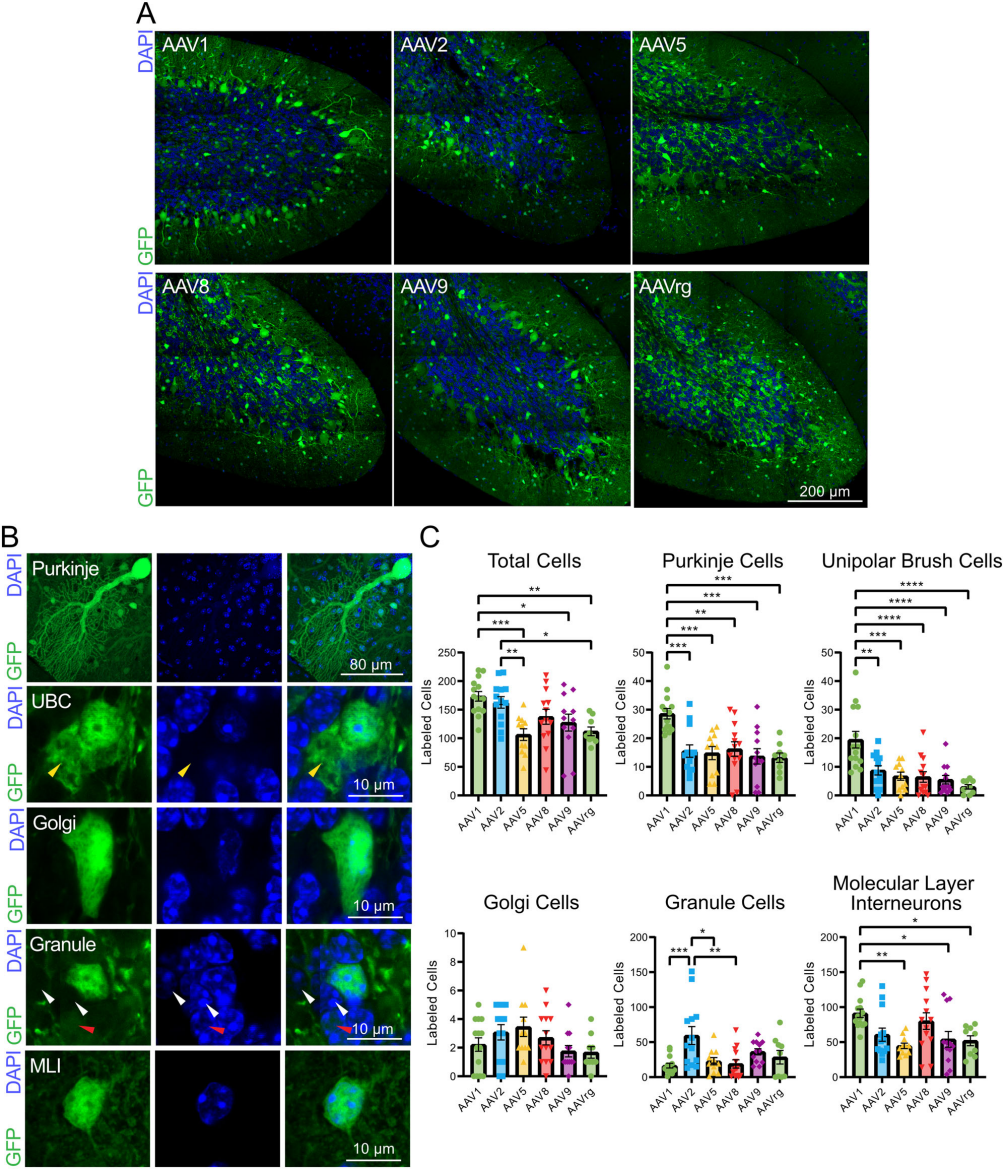
AAV serotype tropism in the cerebellum. ***A***, Representative sagittal sections of lobule X of the cerebellum. ***B***, Representative images of cerebellar cell types. Fluorescent cells were characterized utilizing the unique morphology of cerebellar cortex cell types. The yellow arrow indicates the dendritic brush unique to unipolar brush cells. White arrows indicate DAPI-positive cells devoid of GFP expression and the red arrow indicates a DAPI-positive cell with dim GFP expression. ***C***, Bar graphs showing the number of GFP-positive cerebellar cell types across AAV serotypes. Images magnified to 63× of lobule X of the cerebellum were analyzed to determine tropism of AAV1, 2, 5, 8, 9, and rg. The AAV1 serotype labeled significantly more total cells compared with AAV5, 9, and rg. AAV1 labeled significantly more Purkinje and unipolar brush cells compared with all the other serotypes and significantly more molecular layer interneurons than AAV5, 9, and rg. AAV2 transfected significantly more granule cells compared with AAV1, 5, and 8. Identical imaging parameters and brightness and contrast adjustments were applied to all images in the same panel. Statistical comparisons were made using one-way ANOVAs followed by Tukey's HSD tests. Error bars are SEM. * indicates *p* < 0.05, ** indicates *p* < 0.01, *** indicates *p* < 0.001, **** indicates *p* < 0.0001.

The cerebellum is highly organized with three distinct cortical layers—the granule cell layer, Purkinje cell layer, and molecular layer—and has a host of cell types that can be identified by their location within these layers and by their unique morphologies (see Materials and Methods). The synapsin promoter ensures transgene expression is only driven in neurons (Kügler et al., 2003), thus allowing for visual analysis of cell-type tropism within the cerebellum. High-resolution images of DAPI and GFP in lobule X of the cerebellum were imaged on a confocal microscope (Fig. 3*A*). Fluorescently labeled cells were confirmed to contain a DAPI-stained nucleus and were manually annotated by cell type (Fig. 3*B*).

This analysis revealed that serotypes varied in their ability to transfect different cerebellar cell types. As expected, AAV1 labeled significantly more total cells, although AAV2 also labeled many cells, despite the relatively low labeled volume shown in the previous figure (Fig. 3*C*, Table 3). AAV1 was the most effective across cell types, and labeled significantly more Purkinje and UBCs compared with all the other serotypes, and significantly more molecular layer interneurons compared with AAV5, 9, and rg (Fig. 3*C*). However, AAV2 transfected significantly more granule cells than the other serotypes and reached significance when compared with AAV1, 5, and 8 (Fig. 3*C*). Thus, AAV1 labeled the largest variety of cell types, whereas AAV2 labeled granule cells most effectively.

**Table 3.** Statistics associated with Figure 3

Associated figure	Statistical test	Groups compared	Test statistic	*p* value	Mean difference	*N* (slices)
Fig. 3*C*, total cells	One-way ANOVA	All	*F*_(6,72)_ = 5.771	0.0002		72
Fig. 3*C*, total cells	Tukey's test	AAV1 vs AAV5	*q* = 6.042	0.0009	66.99	25
Fig. 3*C*, total cells	Tukey's test	AAV1 vs AAV9	*q* = 4.243	0.0423	45.94	26
Fig. 3*C*, total cells	Tukey's test	AAV1 vs AAVrg	*q* = 5.209	0.0060	61.25	23
Fig. 3*C*, total cells	Tukey's test	AAV2 vs AAV5	*q* = 5.003	0.0093	56.41	24
Fig. 3*C*, total cells	Tukey's test	AAV2 vs AAVrg	*q* = 4.245	0.0421	50.66	22
Fig. 3*C*, Purkinje cells	One-way ANOVA	All	*F*_(6,72)_ = 7.164	<0.0001		72
Fig. 3*C*, Purkinje cells	Tukey's test	AAV1 vs AAV2	*q* = 6.096	0.0008	13.03	27
Fig. 3*C*, Purkinje cells	Tukey's test	AAV1 vs AAV5	*q* = 6.150	0.0007	13.75	25
Fig. 3*C*, Purkinje cells	Tukey's test	AAV1 vs AAV8	*q* = 5.772	0.0017	12.34	27
Fig. 3*C*, Purkinje cells	Tukey's test	AAV1 vs AAV9	*q* = 6.826	0.0001	14.90	26
Fig. 3*C*, Purkinje cells	Tukey's test	AAV1 vs AAVrg	*q* = 6.472	0.0003	15.35	23
Fig. 3*C*, UBCs	One-way ANOVA	All	*F*_(6,72)_ = 9.435	<0.0001		72
Fig. 3*C*, UBCs	Tukey's test	AAV1 vs AAV2	*q* = 5.830	0.0014	10.73	27
Fig. 3*C*, UBCs	Tukey's test	AAV1 vs AAV5	*q* = 6.634	0.0002	12.77	25
Fig. 3*C*, UBCs	Tukey's test	AAV1 vs AAV8	*q* = 7.084	<0.0001	13.04	27
Fig. 3*C*, UBCs	Tukey's test	AAV1 vs AAV9	*q* = 7.447	<0.0001	14.00	26
Fig. 3*C*, UBCs	Tukey's test	AAV1 vs AAVrg	*q* = 8.082	<0.0001	16.50	23
Fig. 3*C*, Golgi cells	One-way ANOVA	All	*F*_(6,72)_ = 1.847	0.1157		72
Fig. 3*C*, granule cells	One-way ANOVA	All	*F*_(6,72)_ = 4.792	0.0009		72
Fig. 3*C*, granule cells	Tukey's test	AAV1 vs AAV2	*q* = 6.016	0.0009	−43.03	27
Fig. 3*C*, granule cells	Tukey's test	AAV2 vs AAV5	*q* = 4.828	0.0134	36.73	24
Fig. 3*C*, granule cells	Tukey's test	AAV2 vs AAV8	*q* = 5.575	0.0026	40.62	26
Fig. 3*C*, MLIs	One-way ANOVA	All	*F*_(6,72)_ = 4.191	0.0023		72
Fig. 3*C*, MLIs	Tukey's test	AAV1 vs AAV5	*q* = 46.49	0.0055	5.243	25
Fig. 3*C*, MLIs	Tukey's test	AAV1 vs AAV9	*q* = 37.21	0.0381	4.299	26
Fig. 3*C*, MLIs	Tukey's test	AAV1 vs AAVrg	*q* = 39.21	0.0482	4.171	25

### Cerebellar axonal projections

Expression of the AAV-hSyn-GFP transgene leads to cytosolic GFP expression and labeling throughout the entire neuron, including dendrites and axonal projections. Labeling axonal projections is useful to define projection targets of an injected area and is additionally significant to researchers utilizing AAVs to deliver genes encoding axonal proteins. All AAV serotypes labeled axonal projections to various regions distant from the injection site. These projections are evaluated for differences between serotypes below.

**Figure 4.**
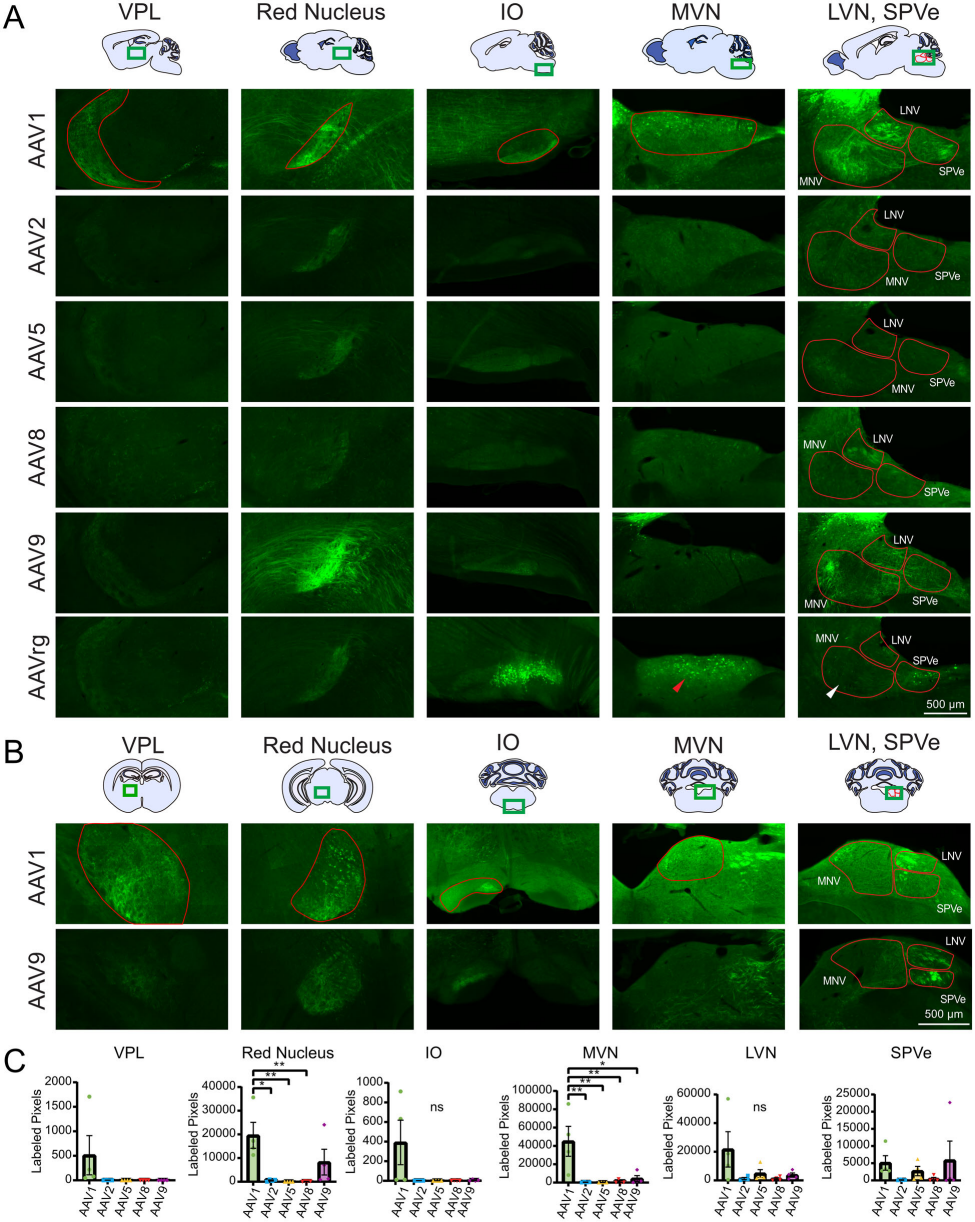
Labeled axonal projections from the cerebellum to the ventral posterolateral nucleus of the thalamus, the red nucleus, the inferior olive, the vestibular nuclei. ***A***, Representative images of the VPL, RN, IO, MVN, SPVe, and LVN by serotype. Differences in the degree of retrograde labeling within the MVN were present for slices infected with AAVrg (red arrow vs white arrow). ***B***, Representative coronal images of contralateral VPL, RN, IO, and the ipsilateral MVN, SPVe, and LVN. ***C***, Bar graphs showing serotype brightness quantification for each region. Labeled projections in all regions could be seen in slices infected AAV1 and AAV9, projections in the contralateral IO, ipsilateral MVN, LVN, and SPVe could be seen in slices infected with AAVrg, and projections in the MVN, LVN, and SPVe could be seen with slices infected with AAV8. Statistical comparisons were made using one-way ANOVAs followed by Tukey's HSD tests. VPL and SPVe were not statistically tested. Error bars are SEM. * indicates *p* < 0.05, ** indicates *p* < 0.01, ns indicates *p* > 0.05.

The thalamus receives axonal input from the cerebellum which is processed and conveyed to the cerebral cortex (Habas et al., 2019). The main targeted region is the ventral posterolateral nucleus of the thalamus (VPL; Asanuma et al., 1983). Comparisons of these observations were made by summing the total number of labeled pixels per region, for each injection, per serotype. Projections to VPL were labeled following injection of AAV1 better than the other serotypes (Fig. 4, Table 4). The red nucleus receives axonal input from the interposed nucleus of the cerebellar nuclei, which has been implicated in skilled reach and locomotor movement (Low et al., 2018; Judd et al., 2021). AAV1 and 9 produced more axonal labeling than did the other serotypes (Fig. 4). These differences could reflect tropism of AAV1 and AAV9 to the interposed nucleus of the cerebellar nuclei.

**Table 4.** Statistics associated with Figure 4

Associated figure	Statistical test	Groups compared	Test statistic	*p* value	Mean difference	*N* (animals)
Fig. 4*C*, VPL	Not tested					
Fig. 4*C*, red nucleus	One-way ANOVA	All	*F*_(5,20)_ = 6.846	0.0024		20
Fig. 4*C*, red nucleus	Tukey's test	AAV1 vs AAV2	*q* = 5.518	0.0105	19,057	8
Fig. 4*C*, red nucleus	Tukey's test	AAV1 vs AAV5	*q* = 5.670	0.0086	19,581	8
Fig. 4*C*, red nucleus	Tukey's test	AAV1 vs AAV8	*q* = 5.631	0.0090	19,449	8
Fig. 4*C*, IO	One-way ANOVA	All	*F*_(4,15)_ = 2.941	0.0559		20
Fig. 4*C*, MVN	One-way ANOVA	All	*F*_(6,72)_ = 6.070	0.0041		20
Fig. 4*C*, MVN	Tukey's test	AAV1 vs AAV2	*q* = 5.979	0.0056	44,654	8
Fig. 4*C*, MVN	Tukey's test	AAV1 vs AAV5	*q* = 6.011	0.0054	44,888	8
Fig. 4*C*, MVN	Tukey's test	AAV1 vs AAV8	*q* = 5.883	0.0064	43,932	8
Fig. 4*C*, MVN	Tukey's test	AAV1 vs AAV9	*q* = 5.440	0.0117	40,628	8
Fig. 4*C*, LVN	One-way ANOVA	All	*F*_(4,15)_ = 2.270	0.1100		20
Fig. 4*C*, SPVe	Not tested					

The inferior olive (IO) is the source of climbing fiber input to the cerebellum and is involved in learning and timing of movements and comparing intended with achieved movements (De Zeeuw et al., 1998). The IO also receives descending input from the cerebellar nuclei. Labeled axonal projections were found in the contralateral IO following injection of serotypes AAV1 (Fig. 4).

The medial vestibular nucleus (MVN), lateral vestibular nucleus (LVN), and the spinal vestibular nucleus (SPVe) are three of the five vestibular nuclei where primary vestibular information is collected, processed, and modified by other sensory inputs (Barmack, 2023). Labeling was seen in the ipsilateral MVN, LVN, and SPVe following injection of AAV1 into the cerebellum (Fig. 4). In the MVN, AAV1 demonstrated significantly more labeling than AAV2, 5, 8, and 9 (Fig. 4). AAV1 had particularly greater labeling in the SPVe compared with AAV2 and 8.

Retrograde labeling was also seen in the ipsilateral vestibular nuclei where 2/8, 1/8, and 7/8 slices contained retrogradely labeled cells by AAV1, 5, and rg, respectively. The mean number of retrogradely labeled cells by AAVrg varied across the mediolateral extent of MVN, as seen in Figure 4*A* (white vs red arrows). The more medial regions of MVN in slices that did not contain LVN and SPVe had many more labeled cells following injection of AAVrg into the cerebellum (Fig. 4*A*, white arrow; mean ± SEM: 51.2 ± 9.3 labeled cells; *n* = 4) compared with more lateral regions of MVN containing all three vestibular nuclei (Fig. 4*A*, red arrow; mean ± SEM: 0.75 ± 0.25 labeled cells; *n* = 4). The observation that different zones of lobule X project to different vestibular nuclei may explain this finding (Wylie et al., 1994). Labeled cells were also observed in the LVN for 2/4 and 1/4 experiments by AAV1 and rg, respectively, while labeled cells in the SPVe were found in 1/4 and 4/4 experiments for AAV8 and rg, respectively. Additional retrograde labeling in other regions is explored below.

### Inferior collicular axonal projections

**Figure 5.**
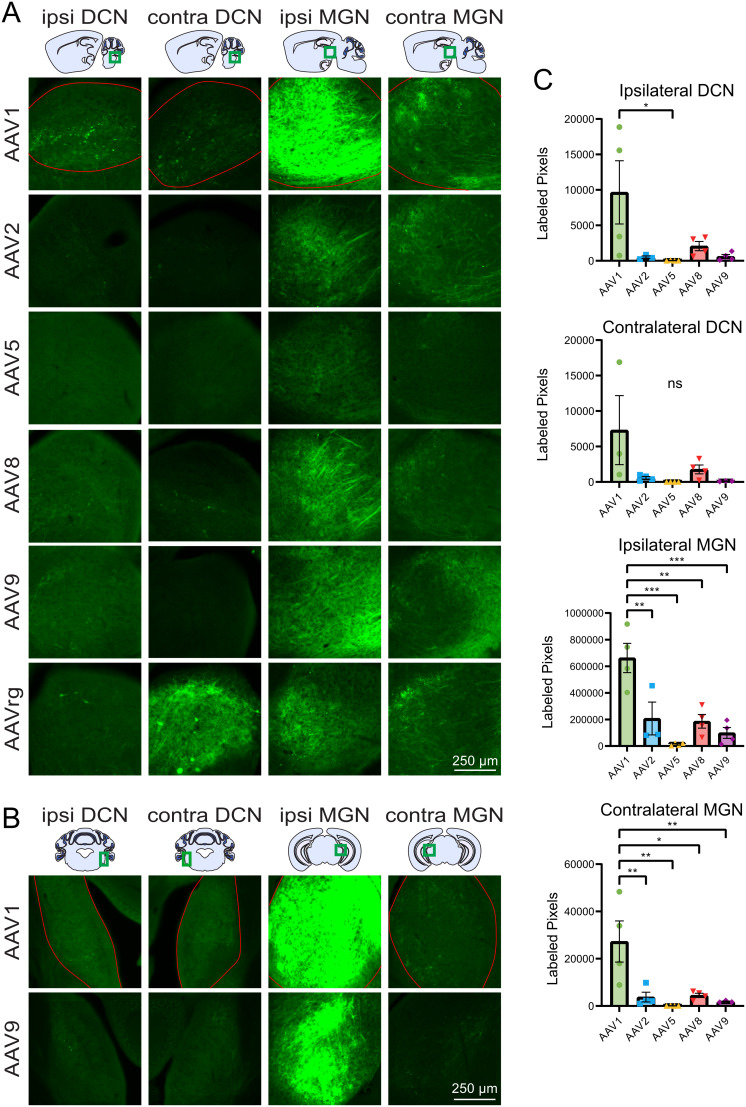
Labeled IC projections to the dorsal cochlear nucleus and medial geniculate nucleus. ***A***, Representative images of the DCN and MGN both ipsilateral and contralateral to the injection site. ***B***, Additional coronal images of the ipsilateral and contralateral DCN and MGN. ***C***, Bar graphs showing the mean serotype brightness quantification for each region. Statistical comparisons were made using one-way ANOVAs followed by Tukey's HSD tests. Error bars are SEM. * indicates *p* < 0.05, ** indicates *p* < 0.01, *** indicates *p* < 0.001, ns indicates *p* > 0.05.

The DCN is a major source of ascending auditory input to the IC and the IC sends a large descending projection back to the DCN (Saldaña, 1993; Milinkeviciute et al., 2017; Balmer and Trussell, 2022). Labeled descending axonal projections were found in the ipsilateral DCN following injection of AAV1 to the IC with little-to-no labeling found following injection of AAV2, 5, 8, and 9; however, AAV1 was the only serotype that was significantly greater than AAV4 with this small sample size (Fig. 5, Table 5). Labeling in the contralateral DCN was seen following injection of AAVrg to the IC and is likely a combination of axonal projections originating from labeled cells in the IC, DCN neurons that project to IC that are retrogradely labeled, and projections originating from retrogradely labeled cells in other brain regions. Meanwhile, sparse axonal labeling within in the contralateral DCN was seen following injection of AAV1 into the IC. Retrograde labeling was seen in the ipsilateral DCN for 2/4 AAVrg experiments, while labeling in the contralateral DCN was seen in 3/3 and 3/4 experiments for AAV1 and AAVrg, respectively.

**Table 5.** Statistics associated with Figure 5

Associated figure	Statistical test	Groups compared	Test statistic	*p* value	Mean difference	*N* (animals)
Fig. 5*C*, Ipsi DCN	One-way ANOVA	All	*F*_(5,19)_ = 3.658	0.0306		19
Fig. 5*C*, Ipsi DCN	Tukey's test	AAV1 vs AAV5	*q* = 4.608	0.0386	9,626	8
Fig. 5*C*, Contra DCN	One-way ANOVA	All	*F*_(5,18)_ = 2.603	0.0850		18
Fig. 5*C*, Ipsi MGN	One-way ANOVA	All	*F*_(5,19)_ = 12.30	0.0002		19
Fig. 5*C*, Ipsi MGN	Tukey's test	AAV1 vs AAV2	*q* = 5.870	0.0073	454,896	7
Fig. 5*C*, Ipsi MGN	Tukey's test	AAV1 vs AAV5	*q* = 9.047	0.0001	649,053	8
Fig. 5*C*, Ipsi MGN	Tukey's test	AAV1 vs AAV8	*q* = 6.644	0.0027	476,635	8
Fig. 5*C*, Ipsi MGN	Tukey's test	AAV1 vs AAV9	*q* = 7.849	0.0006	563,098	8
Fig. 5*C*, Contra MGN	One-way ANOVA	All	*F*_(5,19)_ = 7.106	0.0024		19
Fig. 5*C*, Contra MGN	Tukey's test	AAV1 vs AAV2	*q* = 5.643	0.0099	23,494	8
Fig. 5*C*, Contra MGN	Tukey's test	AAV1 vs AAV5	*q* = 6.533	0.0031	27,197	8
Fig. 5*C*, Contra MGN	Tukey's test	AAV1 vs AAV8	*q* = 5.473	0.0124	22,786	8
Fig. 5*C*, Contra MGN	Tukey's test	AAV1 vs AAV9	*q* = 5.656	0.0097	25,431	7

The main target of the IC is the ipsilateral MGN, which also integrates input from multiple ascending and descending sources (Winer and Schreiner, 2005). Extensive labeling was seen in both the ventral and dorsal divisions of the ipsilateral MGN for all serotypes with brightness decreasing in the following order: AAV1 >>> AAV2 > AAV8 > AAV9 > AAV5. AAV1 produced significantly brighter labeling in the ipsilateral MGN than all other serotypes (Fig. 5*B*). Less intense labeling was seen in the contralateral MGN compared with the ipsilateral MGN following injection of the all the serotypes; however, AAV1 produced significantly greater labeling than AAV2, 5, 8, and 9 (Fig. 5*B*). Across all experiments only one retrogradely labeled cell was found in the ipsilateral MGN following injection of AAVrg into the IC.

### Retrograde labeling of the nucleus of the lateral lemniscus by AAVs injected into the IC

**Figure 6.**
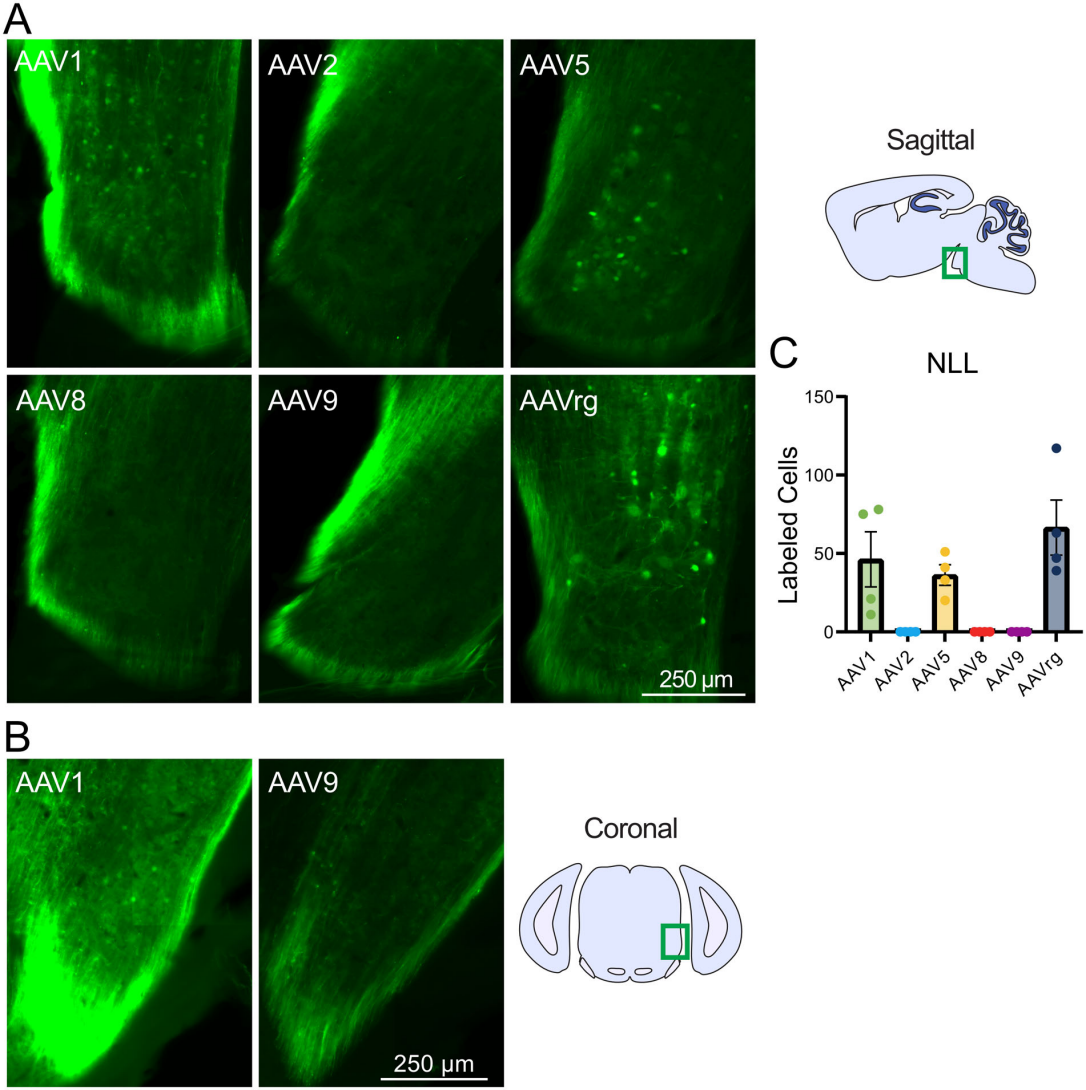
Retrograde labeling of the nucleus of the lateral lemniscus from the IC. ***A***, Representative sagittal images of the ipsilateral nucleus of the lateral lemniscus by serotype. ***B***, Representative coronal images of the nucleus of the lateral lemniscus following AAV1 and 9 injection to the IC. ***C***, Bar graph showing mean number of labeled cells by serotype. Labeled cell bodies were present in the nucleus of the lateral lemniscus in brains injected with AAV1, 5, and rg, but completely absent AAV2, 8, or 9. Identical imaging parameters and brightness and contrast adjustments were applied to all images in the same panel. No statistical tests were performed.

The lateral lemniscus is a fiber bundle that connects the cochlear nuclei and superior olivary complex with the IC (Felmy, 2019). The nucleus of the lateral lemniscus (NLL) is known for its generation of long-lasting inhibition in its contralateral counterpart and the IC (Felmy, 2019). Labeled cell bodies were found in the ipsilateral NLL following injections of AAV1, 5, and rg, indicating retrograde labeling of these cells that project to the IC. As expected, AAVrg retrogradely labeled the greatest number of NLL neurons (Fig. 6). AAV2, 8, and 9 did not retrogradely label any cells in any experiments (Fig. 6). However, AAV1 and AAV5 retrogradely labeled a number of neurons comparable with AAVrg (Fig. 6).

### Retrograde labeling of the cuneate nucleus by AAVs injected into the cerebellum

**Figure 7.**
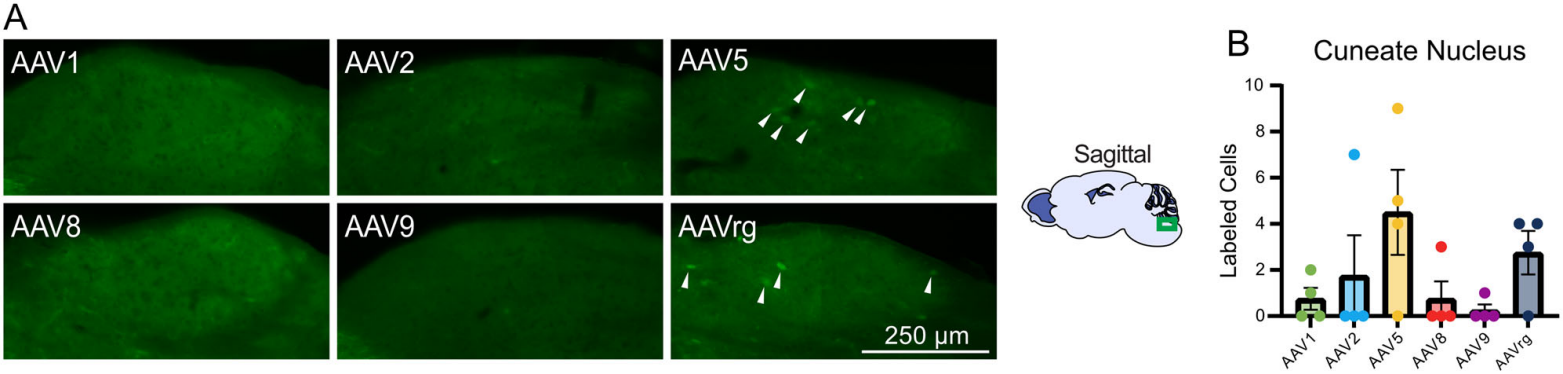
Retrograde labeling of the cuneate nucleus from the cerebellum. ***A***, Representative images of the ipsilateral cuneate nucleus by serotype. ***B***, Bar graph showing mean number of labeled cells by serotype. Labeled cell bodies were present in the cuneate nucleus in brains injected with AAV5 or rg (white arrows). Identical imaging parameters and brightness and contrast adjustments were applied to all images in the same panel. No statistical tests were performed.

The cuneate nucleus is a division of the dorsal column nuclei and receives primary sensory afferents from the upper body and upper limbs (Watson, 2012). AAV5 and AAVrg retrogradely labeled cell bodies in the ipsilateral cuneate nucleus (Fig. 7). Labeled cell bodies were only rarely found in cuneate nucleus following cerebellar injections of other serotypes.

### Summary of labeled axonal projections and retrograde labeling

**Figure 8.**
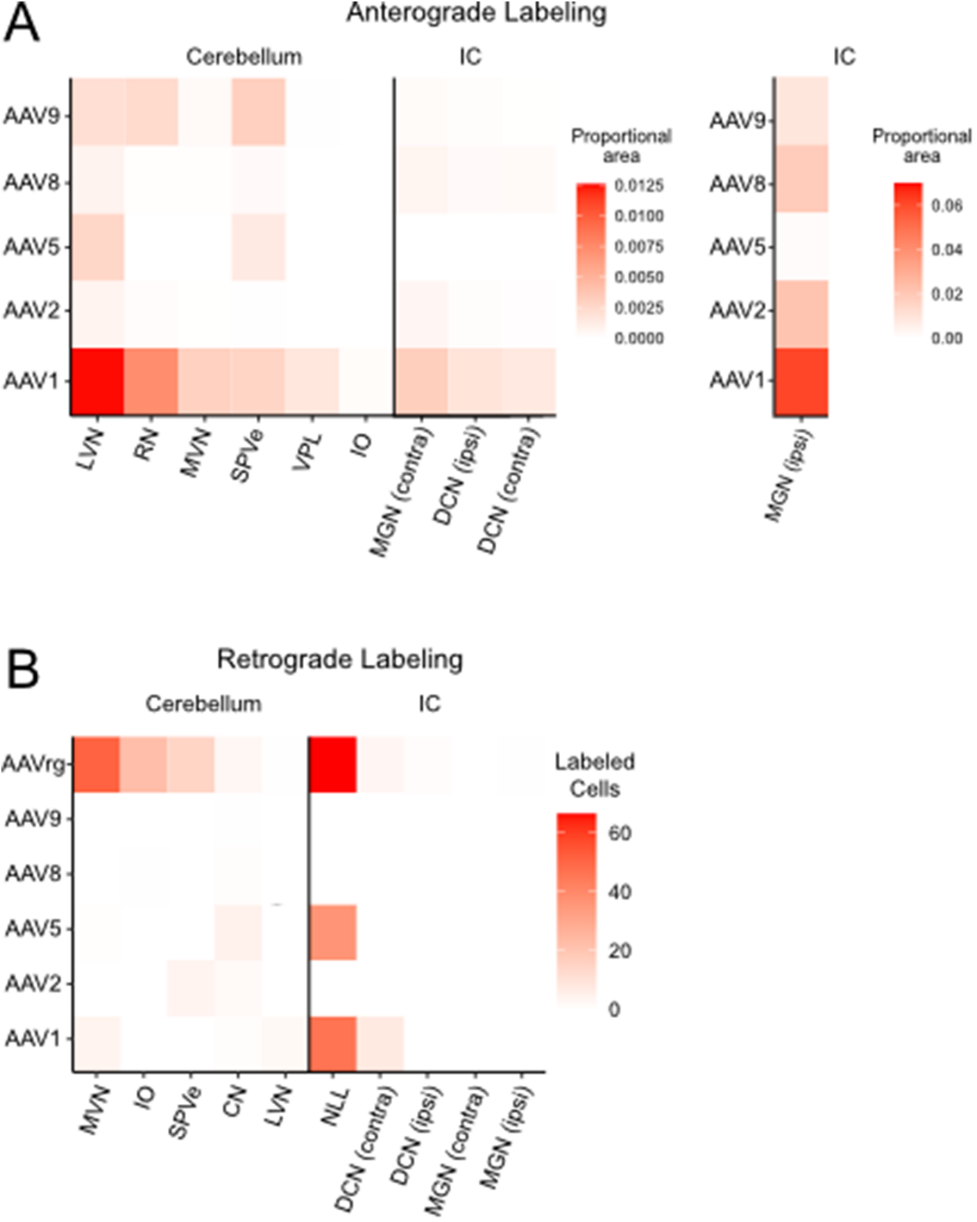
Summary of anterograde and retrograde labeling by serotype across regions of the midbrain and brainstem. Data from the previous figures were normalized to the total area of the nucleus and combined to create heatmaps of (***A***) anterograde and (***B***) retrograde labeling across serotypes and brain areas. Projections from IC to ipsilateral MGN labeled a much larger area than the other projections and are therefore plotted separately using a different color scale. AAV1 produced superior anterograde labeling compared with the other serotypes. As expected, AAVrg produced superior retrograde labeling. Overall, AAV8 and AAV9 had the lowest level of retrograde labeling, while AAV2 and 8 had the lowest anterograde labeling from the cerebellum and AAV5 had the lowest anterograde labeling from the IC.

The degree of retrograde labeling and presence of labeled axonal projections varied between serotypes and brain regions and are summarized in Figure 8. AAV1 labeled the most axonal projections in every area investigated (Fig. 8*A*). All serotypes except AAV9 demonstrated some degree of retrograde labeling in at least one area, with AAVrg having the greatest degree of retrograde labeling, as expected (Fig. 8*B*). The region with the greatest retrograde labeling across multiple serotypes was the nucleus of the lateral lemniscus (Fig. 8*B*).

## Discussion

AAVs are valuable tools to investigate neuronal function and connectivity, and empirical studies are necessary to define their effectiveness in specific brain regions. Such studies allow researchers to choose the optimal serotype to address their experimental questions. Here we provide a systematic characterization of the efficacy of AAV serotypes in the cerebellum and IC, two regions important for multisensory integration and of interest to many researchers. First, we investigated the efficacy of AAV transduction in these regions by examining the extent of fluorescent labeling. Second, tropism within the cerebellum was evaluated by a careful analysis of labeled neurons, which was possible because of their distinct morphologies and localization. Next, differences in the extent of labeling of axonal projections in various targets of the IC and cerebellum were detailed. These differences are presumably due to variations in tropism or transduction efficacy of different populations of projection neurons. Finally, retrograde labeling was described across various input regions to the cerebellum and IC.

We found that AAV1 produces superior transgene expression defined by the number of neurons labeled, brightness of labeling, and volume of tissue labeled, in both the IC and in the cerebellum. Thus, AAV1 may be advantageous to transfect a large volume with a single injection in these regions. On the other hand, experiments requiring highly localized gene expression could benefit from the use of an alternate serotype. In the cerebellum, AAV1 transduced significantly more Purkinje cells and UBCs in lobule X of the cerebellar cortex compared with all other serotypes and transduced significantly more molecular layer interneurons than AAV5, 9, and rg. Meanwhile, AAV2 transduced significantly more granule cells in lobule X of the cerebellar cortex than AAV1, 5, and 8. AAV2 was the most effective serotype for labeling granule cells. Although the labeling was rather dim in granule cells compared with other cell types, this level of gene expression is expected to be sufficient for Cre recombination and other processes that do not require strong overexpression of protein (Haery et al., 2019).

Labeled axonal projections were seen in a variety of IC and cerebellum output regions. Consistently, AAV1 produced the brightest axonal labeling compared with the other serotypes in the MGN, VPL, RN, MVN, ipsilateral DCN, ipsilateral MGN, IO, and LVN, which likely reflects higher transduction efficacy at the injection site or tropism for the specific projection neurons. AAV9 labeled a large number of projections in the red nucleus, which could indicate tropism of cerebellar nuclei neurons that project to this area (Low et al., 2018; Judd et al., 2021). The high degree of labeling in the MVN by AAV1 is likely due to superior transduction of Purkinje cells, whose axons project to MVN (Barmack, 2023).

Superior efficacy of AAV1 has been reported in other brain regions including the cortex, midbrain, corticospinal tract, nigrostriatal system, and red nucleus (C. Wang et al., 2003; Burger et al., 2004; McFarland et al., 2009; Blits et al., 2010; Hutson et al., 2012), while other studies demonstrate a lack of efficacy by AAV2 (Taymans et al., 2007; Aschauer et al., 2013; Watakabe et al., 2015) or higher efficacy of other serotypes (Paterna et al., 2004; Mason et al., 2010; Holehonnur et al., 2014). This region-specific variability of AAV efficacy underscores the necessity of studies such as this one.

All AAVs have been shown to cause some retrograde labeling in various brain regions (Taymans et al., 2007; Hollis et al., 2008; Masamizu et al., 2011; Castle et al., 2014; Tervo et al., 2016; Sun et al., 2019; Haggerty et al., 2020). AAVrg was developed specifically to infect axons and retrogradely label neurons projecting to the area of injection (Tervo et al., 2016). In this study we found retrograde labeling of cells in NLL was higher for AAV1, 5, and rg than the other serotypes tested. AAV8 and 9 produced essentially no retrograde labeling in the regions analyzed and may be useful serotypes when avoiding retrograde labeling is essential.

Enhancing the fluorescence that is present using an immunohistochemical approach would increase the number of neurons and processes that could be visualized, especially fine axonal processes that may have not have been detected here. This is only possible for anatomical studies that use fixed tissue. Higher expression may be necessary for physiological or behavioral studies that use AAVs to express optogenetic or chemogenetic tools or indicators of cellular activity. To increase expression, several modifications to the protocol used here could be made. The 2 week period between injection and analysis that we used is relatively short and longer periods may produce enhanced expression. The titer used here is lower than what is routinely available from commercial sources and a higher titer could increase expression. However, these modifications must be tested, as excessive expression caused by high titers or long incubation periods can cause toxicity (Watakabe et al., 2015).

In this study we only tested AAVs that used the hSyn promoter. A previous study showed that a CaMKII promotor can label Purkinje cells specifically and a GABAA receptor α6 subunit promotor can label granule cells specifically (Kim et al., 2015). Combining the GABAA receptor α6 subunit promotor with the AAV2 serotype may lead to higher expression in granule cells, which are often difficult to label effectively. The present study may provide guidance for neuroscientists planning studies that utilize AAVs to express transgenes in the IC and cerebellum.

## Synthesis

Reviewing Editor: Eran Perlson, Tel Aviv University Decisions are customarily a result of the Reviewing Editor and the peer reviewers coming together and discussing their recommendations until a consensus is reached. When revisions are invited, a fact-based synthesis statement explaining their decision and outlining what is needed to prepare a revision will be listed below. The following reviewer(s) agreed to reveal their identity: Ryota Homma, Marina Silveira.

The manuscript provides valuable insights into the transduction capabilities of different AAV serotypes in two critical brain regions, the inferior colliculus and the cerebellum. Since AAV tropism is known to vary among brain areas and cell types, selecting the appropriate serotype can be challenging without prior screening efforts. The systematic screening performed in this study addresses this challenge, and the results will serve as a useful resource for researchers working in these areas.

The research is rigorously designed and well-executed, providing a solid foundation for advancing the fields of auditory midbrain and cerebellum studies. The data will allow researchers to select the appropriate AAV serotype based on their desired level of viral expression. The clarity of the manuscript makes it easy to follow, still, there are some issues to be addressed:

Major Point

1. It would be helpful to clarify whether the injections were confined to the target regions. For instance, GFP expression in Figure 1B can be seen in the PAG; were these areas excluded from the analysis? Given the goal of validating AAV serotypes, it would be valuable to include more examples showing that injections were restricted to the target regions. Also, clarify whether transfection was biased toward a specific inferior colliculus subdivision and in which subdivision of the MGB (ventral or dorsal) terminals were analyzed.

2. Figure 8A presents heatmaps of pixel counts from earlier figures. However, pixel counts may vary depending on the size of the region of interest (ROI) and do not necessarily reflect the strength of labeling across different structures. Normalizing the data (e.g., dividing by the total number of pixels in the ROI) would help address this issue.

Minor Points

• L92: The statement "(4*10<sup>9</sup> GC total)" likely refers to the titer per injection. Please clarify this explicitly.

• L108: Verify the injection coordinates, as the AP coordinate of the cerebellum seems incorrect.

• L121: The light source used in the Olympus VS200 Slide Scanner should be described, as excitation light intensity can significantly impact fluorescence brightness. Please confirm that the intensity was consistent across all acquisitions.

• L135: Clarify whether the "mean brightness" refers to the mean brightness of pixels above the threshold (20) or the mean of all pixels in the ROI. If it is the former, adding a horizontal line to Figures 1E and 2F at a Mean Pixel Brightness of 20 would indicate the lowest possible value for this measure.

• L167: Prism is the software, and GraphPad is the company. Correct this for clarity.

• L172: Consider mentioning in the Methods that AAVrg is optimized for retrograde labeling, as this information helps contextualize some of the results in subsequent sections.

• L182: Clarify when AAVrg is included in statistical comparisons and when it is excluded, as it is included in some figures.

• L327: Comment on the effectiveness of AAV2 in transducing granule cells when only strong expressions are considered. Is it still more effective than other serotypes?

• Figures 1-2, 4-7: Where appropriate, delineate the ROI in at least one image for each comparison, similar to the rightmost column of Figure 4A/B.

• Figure Legends: If no statistical tests were performed, explicitly state this in the figure legends. This will clarify whether a lack of significance indicates that no test was done or that no significant results were found.

## Author Response

Major Point 1. It would be helpful to clarify whether the injections were confined to the target regions. For instance, GFP expression in Figure 1B can be seen in the PAG; were these areas excluded from the analysis? Given the goal of validating AAV serotypes, it would be valuable to include more examples showing that injections were restricted to the target regions. Also, clarify whether transfection was biased toward a specific inferior colliculus subdivision and in which subdivision of the MGB (ventral or dorsal) terminals were analyzed.

In some cases, cells in the PAG were also labeled. The quantification shown in the manuscript included all the labeled cells including those in the PAG. In order to address this point, we reanalyzed the number of cells labeled within the IC and compared it those labeled outside the IC. We found that restricting the counts to the IC reduced the numbers slightly, but the main conclusions remain the same. We have replaced Figure 1C and 1F with plots using the new values that only include labeled cells within the IC. Interestingly, we found that both AAV1 and AAV9 labeled cells outside the IC at a value of ~10% (of the total labeled cells). The others serotypes labeled cells in a more restricted manner. Thus, although AAV9 labeled far fewer cells in total than AAV1, it labeled the same proportion of cells outside of IC, suggesting that it covered a large volume but transfected fewer cells within that volume. We have clarified this analysis in the Methods section and lines 197-203 in the results section. We thank the reviewer for this comment, as the suggested analysis yielded new information that we would have otherwise overlooked.

The AAV injections were made into the central IC and the volume of 500 nl was sufficient to diffuse to all regions including dorsal and external nuclei of the IC and it did not appear to be biased to one region or another.

In the MGB, both the ventral and dorsal regions had labeled terminals- please see figure below.

2. Figure 8A presents heatmaps of pixel counts from earlier figures. However, pixel counts may vary depending on the size of the region of interest (ROI) and do not necessarily reflect the strength of labeling across different structures. Normalizing the data (e.g., dividing by the total number of pixels in the ROI) would help address this issue.

Thank you for this suggestion. You are correct that normalizing the data so that it represents the proportion of labeled area would reflect the strength of labeling better and not be confounded by the different total size of the areas. We have replaced the figure with the one suggested that is normalized and represents the proportion of the area labeled within a target nucleus.

Minor Points • L92: The statement "(4*109 GC total)" likely refers to the titer per injection. Please clarify this explicitly.

Clarified to "4X10^9 GC per 500 nl injection" • L108: Verify the injection coordinates, as the AP coordinate of the cerebellum seems incorrect.

This was indeed incorrect and has been corrected. • L121: The light source used in the Olympus VS200 Slide Scanner should be described, as excitation light intensity can significantly impact fluorescence brightness. Please confirm that the intensity was consistent across all acquisitions.

The light source was an X-Cite XYLIS broad spectrum LED and the light intensity was maintained at 100% across all imaging. The exposure time was also maintained across experiments, as described. These details have been added to the methods section. • L135: Clarify whether the "mean brightness" refers to the mean brightness of pixels above the threshold (20) or the mean of all pixels in the ROI. If it is the former, adding a horizontal line to Figures 1E and 2F at a Mean Pixel Brightness of 20 would indicate the lowest possible value for this measure.

The mean brightness was only inclusive of pixels above threshold and this has been clarified in the methods section. We also added horizontal lines to figures 1E and 2F as requested. • L167: Prism is the software, and GraphPad is the company. Correct this for clarity.

Corrected • L172: Consider mentioning in the Methods that AAVrg is optimized for retrograde labeling, as this information helps contextualize some of the results in subsequent sections.

We have added this information to the methods section on line 92 as suggested. • L182: Clarify when AAVrg is included in statistical comparisons and when it is excluded, as it is included in some figures.

Statistical comparisons did not include AAVrg except the analysis of cell type tropism in the cerebellar cortex. This has been clarified in the methods section on line 94. • L327: Comment on the effectiveness of AAV2 in transducing granule cells when only strong expressions are considered. Is it still more effective than other serotypes? In all the serotypes there was a range of labeling intensity across the population of labeled granule cells. There were no apparent differences in the proportion of strongly labeled granule cells between serotype and it is clear that AAV2 labeled granule cells more strongly than did the other serotypes, whether considering only the brightest labeled granule cells or all cells with detectable labeling. We added this explanation on line 154. • Figures 1-2, 4-7: Where appropriate, delineate the ROI in at least one image for each comparison, similar to the rightmost column of Figure 4A/B.

Outlines of the areas were added in the first example for Figures 4 and 5. For figures 6 and 7 the region of interest is essentially the entire image so we did not add outlines. For figures 1 and 2 the fluorescence of the entire area was measured across several images, as described above. We believe that the IC and cerebellum will be clear to the reader without outlining them and any confusion should be clarified by the cartoons provided. • Figure Legends: If no statistical tests were performed, explicitly state this in the figure legends. This will clarify whether a lack of significance indicates that no test was done or that no significant results were found. 'ns' was added to figures where a statistical test (ANOVA) resulted in a non-significant difference and these have a been added to the statistical tables as well. The figure legends now clarify if no statistical tests were performed.

## References

[B1] Alisky JM, Hughes SM, Sauter SL, Jolly D, Dubensky TWJ, Staber PD, Chiorini JA, Davidson BL (2000) Transduction of murine cerebellar neurons with recombinant FIV and AAV5 vectors. Neuroreport 11:2669–2673. 10.1097/00001756-200008210-00013 10976941

[B2] Apps R, Garwicz M (2005) Anatomical and physiological foundations of cerebellar information processing. Nat Rev Neurosci 6:297–311. 10.1038/nrn1646 15803161

[B3] Asanuma C, Thach WT, Jones EG (1983) Distribution of cerebellar terminations and their relation to other afferent terminations in the ventral lateral thalamic region of the monkey. Brain Res Rev 5:237–265. 10.1016/0165-0173(83)90015-2 6189561

[B4] Aschauer DF, Kreuz S, Rumpel S (2013) Analysis of transduction efficiency, tropism and axonal transport of AAV serotypes 1, 2, 5, 6, 8 and 9 in the mouse brain (Qiu J, ed). PLoS One 8:e76310. 10.1371/journal.pone.0076310 24086725 PMC3785459

[B5] Atchison RW, Casto BC, Hammon WM (1965) Adenovirus-associated defective virus particles. Science 149:754–756. 10.1126/science.149.3685.754 14325163

[B6] Balmer TS, Trussell LO (2022) Descending axonal projections from the inferior colliculus target nearly all excitatory and inhibitory cell types of the dorsal cochlear nucleus. J Neurosci 42:3381–3393. 10.1523/JNEUROSCI.1190-21.2022 35273085 PMC9034789

[B7] Barmack NH (2023) Vestibular nuclei and their cerebellar connections. In: *Essentials of cerebellum and cerebellar disorders* (Gruol DL, Koibuchi N, Manto M, Molinari M, Schmahmann JD, Shen Y, eds), pp 51–59. Cham: Springer International Publishing.

[B8] Blits B, Derks S, Twisk J, Ehlert E, Prins J, Verhaagen J (2010) Adeno-associated viral vector (AAV)-mediated gene transfer in the red nucleus of the adult rat brain: comparative analysis of the transduction properties of seven AAV serotypes and lentiviral vectors. J Neurosci Methods 185:257–263. 10.1016/j.jneumeth.2009.10.009 19850079

[B9] Bosch MK, Nerbonne JM, Ornitz DM (2014) Dual transgene expression in murine cerebellar Purkinje neurons by viral transduction in vivo (Gonzalez-Alegre P, ed). PLoS One 9:e104062. 10.1371/journal.pone.0104062 25093726 PMC4122438

[B10] Broekman MLD, Comer LA, Hyman BT, Sena-Esteves M (2006) Adeno-associated virus vectors serotyped with AAV8 capsid are more efficient than AAV-1 or -2 serotypes for widespread gene delivery to the neonatal mouse brain. Neuroscience 138:501–510. 10.1016/j.neuroscience.2005.11.057 16414198

[B11] Burger C, Gorbatyuk OS, Velardo MJ, Peden CS, Williams P, Zolotukhin S, Reier PJ, Mandel RJ, Muzyczka N (2004) Recombinant AAV viral vectors pseudotyped with viral capsids from serotypes 1, 2, and 5 display differential efficiency and cell tropism after delivery to different regions of the central nervous system. Mol Ther 10:302–317. 10.1016/j.ymthe.2004.05.024 15294177

[B12] Castle MJ, Gershenson ZT, Giles AR, Holzbaur ELF, Wolfe JH (2014) Adeno-associated virus serotypes 1, 8, and 9 share conserved mechanisms for anterograde and retrograde axonal transport. Hum Gene Ther 25:705–720. 10.1089/hum.2013.189 24694006 PMC4137353

[B13] Cearley CN, Wolfe JH (2006) Transduction characteristics of adeno-associated virus vectors expressing cap serotypes 7, 8, 9, and Rh10 in the mouse brain. Mol Ther 13:528–537. 10.1016/j.ymthe.2005.11.015 16413228

[B14] De Zeeuw CI, Hoogenraad CC, Koekkoek SKE, Ruigrok TJH, Galjart N, Simpson JI (1998) Microcircuitry and function of the inferior olive. Trends Neurosci 21:391–400. 10.1016/S0166-2236(98)01310-1 9735947

[B15] De Zeeuw CI, Lisberger SG, Raymond JL (2021) Diversity and dynamism in the cerebellum. Nat Neurosci 24:160–167. 10.1038/s41593-020-00754-9 33288911

[B16] El-Shamayleh Y, Kojima Y, Soetedjo R, Horwitz GD (2017) Selective optogenetic control of Purkinje cells in monkey cerebellum. Neuron 95:51–62.e4. 10.1016/j.neuron.2017.06.002 28648497 PMC5547905

[B17] Felmy F (2019) The nuclei of the lateral lemniscus. In: *The Oxford handbook of the auditory brainstem* (Kandler K, ed), Ed 1. pp 445–472. Oxford, United Kingdom: Oxford University Press.

[B18] Habas C, Manto M, Cabaraux P (2019) The cerebellar thalamus. Cerebellum 18:635–648. 10.1007/s12311-019-01019-3 30827014

[B19] Haery L, et al. (2019) Adeno-associated virus technologies and methods for targeted neuronal manipulation. Front Neuroanat 13:93. 10.3389/fnana.2019.00093 31849618 PMC6902037

[B20] Haggerty DL, Grecco GG, Reeves KC, Atwood B (2020) Adeno-associated viral vectors in neuroscience research. Mol Ther Methods Clin Dev 17:69–82. 10.1016/j.omtm.2019.11.012 31890742 PMC6931098

[B21] Hirai H (2008) Progress in transduction of cerebellar Purkinje cells in vivo using viral vectors. Cerebellum 7:273–278. 10.1007/s12311-008-0012-5 18418690

[B22] Hoggan MD, Blacklow NR, Rowe WP (1966) Studies of small DNA viruses found in various adenovirus preparations: physical, biological, and immunological characteristics. Proc Natl Acad Sci U S A 55:1467–1474. 10.1073/pnas.55.6.1467 5227666 PMC224346

[B23] Holehonnur R, Luong JA, Chaturvedi D, Ho A, Lella SK, Hosek MP, Ploski JE (2014) Adeno-associated viral serotypes produce differing titers and differentially transduce neurons within the rat basal and lateral amygdala. BMC Neurosci 15:28. 10.1186/1471-2202-15-28 24533621 PMC3937004

[B24] Hollis ER, Kadoya K, Hirsch M, Samulski RJ, Tuszynski MH (2008) Efficient retrograde neuronal transduction utilizing self-complementary AAV1. Mol Ther 16:296–301. 10.1038/sj.mt.6300367 18223548

[B25] Hutson TH, Verhaagen J, Yáñez-Muñoz RJ, Moon LDF (2012) Corticospinal tract transduction: a comparison of seven adeno-associated viral vector serotypes and a non-integrating lentiviral vector. Gene Ther 19:49–60. 10.1038/gt.2011.71 21562590 PMC3160493

[B26] Jackman SL, Beneduce BM, Drew IR, Regehr WG (2014) Achieving high-frequency optical control of synaptic transmission. J Neurosci 34:7704–7714. 10.1523/JNEUROSCI.4694-13.2014 24872574 PMC4035530

[B27] Judd EN, Lewis SM, Person AL (2021) Diverse inhibitory projections from the cerebellar interposed nucleus. Elife 10:e66231. 10.7554/eLife.66231 34542410 PMC8483738

[B28] Kaemmerer WF, Reddy RG, Warlick CA, Hartung SD, McIvor RS, Low WC (2000) In vivo transduction of cerebellar Purkinje cells using adeno-associated virus vectors. Mol Ther 2:446–457. 10.1006/mthe.2000.0134 11082318

[B29] Kim Y, Kim T, Rhee JK, Lee D, Tanaka-Yamamoto K, Yamamoto Y (2015) Selective transgene expression in cerebellar Purkinje cells and granule cells using adeno-associated viruses together with specific promoters. Brain Res 1620:1–16. 10.1016/j.brainres.2015.05.015 25988836

[B30] Klein RL, Dayton RD, Tatom JB, Henderson KM, Henning PP (2008) AAV8, 9, Rh10, Rh43 vector gene transfer in the rat brain: effects of serotype, promoter and purification method. Mol Ther 16:89–96. 10.1038/sj.mt.6300331 17955025 PMC2987640

[B31] Kügler S, Kilic E, Bähr M (2003) Human synapsin 1 gene promoter confers highly neuron-specific long-term transgene expression from an adenoviral vector in the adult rat brain depending on the transduced area. Gene Ther 10:337–347. 10.1038/sj.gt.3301905 12595892

[B32] Low AYT, Thanawalla AR, Yip AKK, Kim J, Wong KLL, Tantra M, Augustine GJ, Chen AI (2018) Precision of discrete and rhythmic forelimb movements requires a distinct neuronal subpopulation in the interposed anterior nucleus. Cell Rep 22:2322–2333. 10.1016/j.celrep.2018.02.017 29490269

[B33] Lv J, et al. (2024) AAV1-hOTOF gene therapy for autosomal recessive deafness 9: a single-arm trial. Lancet 403:2317–2325. 10.1016/S0140-6736(23)02874-X 38280389

[B34] Masamizu Y, Okada T, Kawasaki K, Ishibashi H, Yuasa S, Takeda S, Hasegawa I, Nakahara K (2011) Local and retrograde gene transfer into primate neuronal pathways via adeno-associated virus serotype 8 and 9. Neuroscience 193:249–258. 10.1016/j.neuroscience.2011.06.080 21782903

[B35] Mason MR, Ehlert EM, Eggers R, Pool CW, Hermening S, Huseinovic A, Timmermans E, Blits B, Verhaagen J (2010) Comparison of AAV serotypes for gene delivery to dorsal root ganglion neurons. Mol Ther 18:715–724. 10.1038/mt.2010.19 20179682 PMC2862541

[B36] McFarland NR, Lee J, Hyman BT, McLean PJ (2009) Comparison of transduction efficiency of recombinant AAV serotypes 1, 2, 5, and 8 in the rat nigrostriatal system. J Neurochem 109:838–845. 10.1111/j.1471-4159.2009.06010.x 19250335 PMC2698947

[B37] Milinkeviciute G, Muniak MA, Ryugo DK (2017) Descending projections from the inferior colliculus to the dorsal cochlear nucleus are excitatory. J Comp Neurol 525:773–793. 10.1002/cne.24095 27513294

[B38] Paterna J-C, Feldon J, Büeler H (2004) Transduction profiles of recombinant adeno-associated virus vectors derived from serotypes 2 and 5 in the nigrostriatal system of rats. J Virol 78:6808–6817. 10.1128/JVI.78.13.6808-6817.2004 15194756 PMC421643

[B39] Reeber SL, Otis TS, Sillitoe RV (2013) New roles for the cerebellum in health and disease. Front Syst Neurosci 7:83. 10.3389/fnsys.2013.00083 24294192 PMC3827539

[B40] Saldaña E (1993) Descending projections from the inferior colliculus to the cochlear nuclei in mammals. In: *The mammalian cochlear nuclei: organization and function* (Merchán MA, Juiz JM, Godfrey DA, Mugnaini E, eds), pp 153–165. Boston, MA: Springer US; NATO ASI series.

[B41] Schindelin J, et al. (2012) Fiji: an open-source platform for biological-image analysis. Nat Methods 9:676–682. 10.1038/nmeth.2019 22743772 PMC3855844

[B42] Snyder RO, Moullier P (2011) *Adeno-associated virus: methods and protocols*. Totowa, NJ: Humana Press.

[B43] Sun L, et al. (2019) Differences in neurotropism and neurotoxicity among retrograde viral tracers. Mol Neurodegener 14:8. 10.1186/s13024-019-0308-6 30736827 PMC6368820

[B44] Taymans J-M, Vandenberghe LH, Haute CVD, Thiry I, Deroose CM, Mortelmans L, Wilson JM, Debyser Z, Baekelandt V (2007) Comparative analysis of adeno-associated viral vector serotypes 1, 2, 5, 7, and 8 in mouse brain. Hum Gene Ther 18:195–206. 10.1089/hum.2006.178 17343566

[B45] Tervo DGR, et al. (2016) A designer AAV variant permits efficient retrograde access to projection neurons. Neuron 92:372–382. 10.1016/j.neuron.2016.09.021 27720486 PMC5872824

[B46] Wang D, Tai PWL, Gao G (2019) Adeno-associated virus vector as a platform for gene therapy delivery. Nat Rev Drug Discov 18:358–378. 10.1038/s41573-019-0012-9 30710128 PMC6927556

[B47] Wang C, Wang C-M, Clark KR, Sferra TJ (2003) Recombinant AAV serotype 1 transduction efficiency and tropism in the murine brain. Gene Ther 10:1528–1534. 10.1038/sj.gt.3302011 12900769

[B48] Watakabe A, Ohtsuka M, Kinoshita M, Takaji M, Isa K, Mizukami H, Ozawa K, Isa T, Yamamori T (2015) Comparative analyses of adeno-associated viral vector serotypes 1, 2, 5, 8 and 9 in marmoset, mouse and macaque cerebral cortex. Neurosci Res 93:144–157. 10.1016/j.neures.2014.09.002 25240284

[B49] Watson C (2012) The somatosensory system. In: *The mouse nervous system* (Watson C, Paxinos G, Puelles L, eds), pp 563–570. Amsterdam: Elsevier.

[B50] Winer JA, Schreiner C (2005) *The inferior colliculus: with 168 illustrations*. New York, NY: Springer.

[B51] Wylie DR, De Zeeuw CI, Digiorgi PL, Simpson JI (1994) Projections of individual Purkinje cells of identified zones in the ventral nodulus to the vestibular and cerebellar nuclei in the rabbit. J Comp Neurol 349:448–463. 10.1002/cne.903490309 7852635

[B52] Xue Y, et al. (2023) RNA base editing therapy cures hearing loss induced by OTOF gene mutation. Mol Ther 31:3520–3530. 10.1016/j.ymthe.2023.10.019 37915172 PMC10727966

[B53] Yizhar O, Fenno LE, Davidson TJ, Mogri M, Deisseroth K (2011) Optogenetics in neural systems. Neuron 71:9–34. 10.1016/j.neuron.2011.06.004 21745635

[B54] Zincarelli C, Soltys S, Rengo G, Rabinowitz JE (2008) Analysis of AAV serotypes 1–9 mediated gene expression and tropism in mice after systemic injection. Mol Ther 16:1073–1080. 10.1038/mt.2008.76 18414476

